# Supplementary material for: Persistent homology of unweighted complex networks via discrete Morse theory
Source: Sci Rep. 2019 Sep 25;9:13817. doi: 10.1038/s41598-019-50202-3 (PMC6761140; doi:10.1038/s41598-019-50202-3)
Supplement: Supplementary file 1 — Appendix [file 41598_2019_50202_MOESM1_ESM.pdf]

Appendix
for
Persistent homology of unweighted complex networks via discrete Morse theory

Harish Kannan[1], **Emil Saucan**[2,3], **Indrava Roy**[1,*], **and Areejit Samal**[1,4,*]

[1]The Institute of Mathematical Sciences (IMSc), Homi Bhabha National Institute (HBNI), Chennai 600113 India
[2]Department of Applied Mathematics, ORT Braude College, Karmiel 2161002 Israel
[3]Department of Electrical Engineering, Technion, Israel Institute of Technology, Haifa 3200003 Israel
[4]Max Planck Institute for Mathematics in the Sciences, Leipzig 04103 Germany
[*]indrava@imsc.res.in; asamal@imsc.res.in

Homology of a simplicial complex

In this section, we provide an overview of the mathematical theory of homology groups of a simplicial complex.

We first describe a mathematical group which provides the machinery to represent paths in a simplicial complex. The p-chain group C_p of a simplicial complex K is the Abelian group generated by the oriented p-simplices in K with coefficients in a field $\mathbb{F}$. Elements c of the p-chain group C_p are referred to as p-chains and have the form $c = \sum_i n_i \alpha_i$ where we use the same notation to represent both the oriented p-simplex and its corresponding generator in C_p and n_i are scalars from the field $\mathbb{F}$. Note that the identity element 0 in C_p is the unique p-chain for which all the coefficients n_i are zero in $\mathbb{F}$. Note also that if two p-simplices, α and β, have the same vertex set but opposite orientations, then the generators of C_p corresponding to the two simplices are inverse of each other (i.e., $\alpha = -\beta$). In figure 1 of the main text, the cycle of length 4 formed by the edges or 1-simplices, $[v_1, v_2]$, $[v_2, v_5]$, $[v_5, v_7]$ and $[v_1, v_7]$, can be represented as an element of the 1-chain group which is $c = [v_1, v_2] + [v_2, v_5] + [v_5, v_7] - [v_1, v_7]$.

The boundary operator ∂_p on the generator corresponding to an oriented p-simplex $\alpha = [v_0, v_1, \cdots, v_p]$ is defined as follows[1]:

$$\partial_p(\alpha) = \sum_{i=0}^{p} (-1)^i [v_0, v_1, \cdots, \hat{v}_i, \cdots, v_p], \tag{A.1}$$

where $\hat{v}_i$ refers to the absence of v_i in the $(p-1)$-simplex. By linear extension, the boundary operator ∂_p on any element $c = \sum_i n_i \alpha_i$ of p-chain group C_p gives:

$$\partial_p(c) = \sum_i n_i \partial_p(\alpha_i) \tag{A.2}$$

Note that the boundary operator in the above equation maps a p-chain to a $(p-1)$-chain. In figure 1 of the main text, the boundary operator applied to the 1-chain $c = [v_0, v_1]$ representing an edge gives $v_1 - v_0$ while the boundary operator applied to the 1-chain $c = [v_1, v_2] + [v_2, v_5] + [v_5, v_7] - [v_1, v_7]$ representing a cycle of length 4 gives 0.

This motivates the definition of p-cycles Z_p and p-boundaries B_p. The p-cycles Z_p are the elements of the p-chain group C_p which are mapped to 0 by the boundary operator ∂_p, and thus:

$$Z_p = \ker(\partial_p) = \{c \in C_p \mid \partial_p(c) = 0\}. \tag{A.3}$$

The p-boundaries B_p is defined as follows:

$$B_p = \mathrm{img}(\partial_{p+1}) = \{c \in C_p \mid \exists\, b \in C_{p+1}, \partial_{p+1}(b) = c\}. \tag{A.4}$$

Thus, p-boundaries B_p are the elements of the p-chain group C_p which also happen to be the boundary of an element in the $(p+1)$-chain group C_{p+1}. Note that both Z_p and B_p are subgroups of the p-chain group C_p. It can be shown that the composition $\partial_p \circ \partial_{p+1} = 0$. This implies that B_p is a subgroup of Z_p[1]. Simply stated, the p-boundary operator ∂_p when applied on a p-boundary gives 0. Hence, it follows that every p-boundary is a p-cycle but not necessarily vice versa.

The p-homology group is defined as[1]:

$$H_p = Z_p / B_p, \tag{A.5}$$

where Z_p/B_p is the quotient group[2] of Z_p over B_p. We informally refer to the elements of p-homology group H_p as p-holes. To provide an intuition for the definition of homology groups, a natural way to describe a p-hole would be to characterize it as a p-cycle which is not a p-boundary. In figure 1 of the main text, the 1-cycle $c = [v_1, v_2] + [v_2, v_5] + [v_5, v_7] - [v_1, v_7]$ is a 1-hole as it is not a 1-boundary of any 2-chain while the 1-cycle $c = [v_2, v_3] + [v_3, v_4] + [v_4, v_2]$ is not a 1-hole as it is the 1-boundary of the 2-simplex $[v_2, v_3, v_4]$. Thus, the concept of quotient groups provide the mathematical machinery to characterize such p-holes in simplicial complexes.

We remark that though it is customary to call H_p a group, since we use coefficients from a field $\mathbb{F}$, it satisfies additional properties which make it a vector space over $\mathbb{F}$. The p-Betti number is defined as the dimension of the homology group H_p viewed as a vector space over field $\mathbb{F}$[1]. Informally, the p-Betti number β_p represents the number of p-holes of the simplicial complex. We remark that the Euler characteristic of the clique complex K with dimension d corresponding to a graph G is given by the alternating sum of Betti numbers[1], namely,

$$\chi(K) = \beta_0 - \beta_1 + \beta_2 - \cdots + (-1)^d \beta_d. \tag{A.6}$$

In this work, we use the finite field $\mathbb{F} = \mathbb{Z}/2$, i.e., the field with two elements.

Proof of concept for Algorithm 1

In this section, we present a rigorous proof for the following theorem.

Theorem 1. *Algorithm 1 produces a discrete Morse function f on any simplicial complex K of finite dimension d.*

Proof. Let K denote a simplicial complex of dimension d. Recall that for the function f on K which is constructed by algorithm 1, for each simplex $\alpha^p \in K$, the two sets U_α and V_α are defined as follows:

$$U_\alpha = \{\beta^{p+1} \mid \alpha^p < \beta^{p+1} \text{ and } f(\beta) \leq f(\alpha)\}$$
$$V_\alpha = \{\gamma^{p-1} \mid \gamma^{p-1} < \alpha^p \text{ and } f(\alpha) \leq f(\gamma)\}$$

To prove that f is a discrete Morse function we need to show that for each simplex $\alpha \in K$, both $|V_\alpha| \leq 1$ and $|U_\alpha| \leq 1$.

Firstly, we show that for each simplex $\alpha \in K$, $|V_\alpha| \leq 1$. Consider a 0-simplex $\alpha \in K$. Since, the dimension of a simplex cannot be less than 0, for each 0-simplex $\alpha \in K$ the corresponding set V_α is empty. In other words, for each 0-simplex $\alpha \in K$, we have that $|V_\alpha| = 0$. Also, for each p-simplex $\alpha^p \in K$ such that $1 \leq p \leq d$, Lemma 6 below shows that $|V_\alpha| \leq 1$. Thus, for every simplex $\alpha \in K$, we have shown that $|V_\alpha| \leq 1$.

Secondly, we show that for each simplex $\alpha \in K$, $|U_\alpha| \leq 1$. For each p-simplex $\alpha^p \in K$ such that $0 \leq p \leq (d-1)$, we prove in Lemma 5 below that $|U_\alpha| \leq 1$. Now consider a d-simplex $\alpha^d \in K$. Since, by assumption K is a d-dimensional simplicial complex, there are no $(d+1)$-simplices in K, and thus, the set U_α for each d-simplex α^d in K is empty. In other words, for each d-simplex $\alpha^d \in K$, we have that $|U_\alpha| = 0$. Thus, for every simplex $\alpha \in K$ we have shown that $|U_\alpha| \leq 1$.

In summary, we have shown that for each simplex $\alpha \in K$, $|V_\alpha| \leq 1$ and $|U_\alpha| \leq 1$. Thus, f satisfies the definition of a discrete Morse function on the simplicial complex K.

□

We next prove the Lemmas used in the proof of the theorem above. This is done in the following sequence of Lemmas 2 to 6. We assume that K is a d-dimensional simplicial complex and f is the output function on K obtained from algorithm 1. We *remark* that a p-dimensional simplex α of a simplicial complex K is denoted by $\alpha^p \in K$. Also, if p-simplex α is a face of a $(p+1)$-simplex β then this is represented as $\alpha^p < \beta^{p+1}$ in the sequel.

Lemma 2. *For each p where $0 \leq p \leq (d-1)$, if $\alpha^p \in K$ and $\beta^{p+1} \in K$ such that $\alpha^p < \beta^{p+1}$, then, $f(\alpha) \neq f(\beta)$.*

Proof. Let $\gamma_0, \gamma_1, \cdots, \gamma_{p+1}$ denote the p-dimensional faces of β^{p+1} such that $f(\gamma_0) \geq f(\gamma_1) \geq \cdots \geq f(\gamma_{p+1})$. Note that α^p is one such γ_i since by assumption α^p is a p-dimensional face of β^{p+1}. Based on lines 11-23 in algorithm 1, we have that:

$$f(\beta) = \begin{cases} (f(\gamma_0) + f(\gamma_1))/2 & \text{if } \texttt{Flag}[\gamma_0] = 0 \text{ and } f(\gamma_0) > f(\gamma_1) \quad \text{(Case } A\text{)} \\ f(\gamma_0) + \varepsilon & \text{otherwise} \quad \text{(Case } B\text{)} \end{cases} \tag{A.7}$$

where $\varepsilon > 0$.
Case A implies $f(\gamma_0) > f(\beta) > f(\gamma_1) \geq f(\gamma_2) \cdots \geq f(\gamma_p) \geq f(\gamma_{p+1})$.
Case B implies $f(\beta) > f(\gamma_0) \geq f(\gamma_1) \geq f(\gamma_2) \cdots \geq f(\gamma_p) \geq f(\gamma_{p+1})$.
Thus, for both cases we have that $f(\beta) \neq f(\gamma_i)$ for each $i \in \{0,1,2,\cdots,(p+1)\}$. Since α^p is one such γ_i for some $i \in \{0,1,2,\cdots,(p+1)\}$, we have $f(\alpha) \neq f(\beta)$.

□

Lemma 3. *For each p where $0 \leq p \leq (d-1)$, if $\alpha^p \in K$ and $\beta^{p+1} \in K$ such that $\alpha^p < \beta^{p+1}$, then $f(\alpha) > f(\beta)$ if and only if $\texttt{Flag}[\alpha]$ changes value from 0 to 1 while assigning function value for β.*

Proof. Given $\alpha^p \in K$ and $\beta^{p+1} \in K$ such that $\alpha^p < \beta^{p+1}$, we first assume $f(\alpha) > f(\beta)$. Let $\gamma_0, \gamma_1, \cdots, \gamma_{p+1}$ denote the p-dimensional faces of β^{p+1} such that $f(\gamma_0) \geq f(\gamma_1) \geq \cdots \geq f(\gamma_{p+1})$. Then, the value $f(\beta)$ is given by equation A.7.
Case A in equation A.7 implies $f(\gamma_0) > f(\beta) > f(\gamma_1) \geq f(\gamma_2) \cdots \geq f(\gamma_p) \geq f(\gamma_{p+1})$.
Case B in equation A.7 implies $f(\beta) > f(\gamma_0) \geq f(\gamma_1) \geq f(\gamma_2) \cdots \geq f(\gamma_p) \geq f(\gamma_{p+1})$.

Since by assumption, α^p is a face of β^{p+1} and $f(\alpha) > f(\beta)$, Case A is applicable, and we have γ_0 equals α^p. Thus, based on line 18 in algorithm 1, while assigning the function value on β^{p+1}, $\texttt{Flag}[\alpha]$ changes value from 0 to 1.

Now, given $\alpha^p \in K$ and $\beta^{p+1} \in K$ such that $\alpha^p < \beta^{p+1}$, we assume that `Flag`$[\alpha]$ changes value from 0 to 1 while assigning function value for β^{p+1}. Let $\gamma_0, \gamma_1, \cdots, \gamma_{p+1}$ denote the p-dimensional faces of β^{p+1} such that $f(\gamma_0) \geq f(\gamma_1) \cdots \geq f(\gamma_{p+1})$. Based on lines 11-23 in algorithm 1, `Flag`$[\alpha]$ changes value from 0 to 1 while assigning function value on β^{p+1} implies that γ_0 equals α^p. Thus, we have that $f(\alpha) > f(\beta) = (f(\alpha) + f(\gamma_1))/2$.

□

Lemma 4. *Let α be a simplex of K. Then, the number of times* `Flag`$[\alpha]$ *changes value from* 0 *to* 1 *is* ≤ 1.

Proof. `Flag`$[\alpha]$ is initially set to 0 in algorithm 1. In algorithm 1, if `Flag`$[\alpha]$ transitions to 1, its value never changes. In other words, there is no procedure in our algorithm 1 which changes the `Flag` of a simplex from 1 to 0. Thus, either `Flag`$[\alpha]$ remains 0 throughout or changes value from 0 to 1 exactly once in algorithm 1.

□

Lemma 5. *For each p where $0 \leq p \leq (d-1)$, if $\alpha^p \in K$, then $|U_\alpha| \leq 1$.*

Proof. From Lemma 3, we have that for each $\alpha^p \in K$, the number of $(p+1)$-simplices β^{p+1} such that $\alpha^p < \beta^{p+1}$ and $f(\beta) < f(\alpha)$ is equal to the number of times `Flag`$[\alpha]$ changes value from 0 to 1. Applying Lemma 4, we get that for each $\alpha^p \in K$,

$$\#\{\beta^{p+1} \mid \alpha^p < \beta^{p+1} \text{ and } f(\beta) < f(\alpha)\} \leq 1.$$

Furthermore, Lemma 2 tells us that,

$$\#\{\beta^{p+1} \mid \alpha^p < \beta^{p+1} \text{ and } f(\beta) < f(\alpha)\} = \#\{\beta^{p+1} \mid \alpha^p < \beta^{p+1} \text{ and } f(\beta) \leq f(\alpha)\}$$

Thus, we have that for each $\alpha^p \in K$, $|U_\alpha| = \#\{\beta^{p+1} \mid \alpha^p < \beta^{p+1} \text{ and } f(\beta) \leq f(\alpha)\} \leq 1$.

□

Lemma 6. *For each p where $1 \leq p \leq d$, if $\alpha^p \in K$, then $|V_\alpha| \leq 1$.*

Proof. Let $\gamma_0, \gamma_1, \cdots, \gamma_p$ denote the $(p-1)$-dimensional faces of $\alpha^p \in K$ such that $f(\gamma_0) \geq f(\gamma_1) \geq \cdots \geq f(\gamma_p)$. Based on lines 11-23 in algorithm 1, we have that:

$$f(\alpha) = \begin{cases} (f(\gamma_0) + f(\gamma_1))/2 & \text{if } \texttt{Flag}[\gamma_0] = 0 \text{ and } f(\gamma_0) > f(\gamma_1) \quad \text{(Case } A) \\ f(\gamma_0) + \varepsilon & \text{otherwise} \quad \text{(Case } B) \end{cases}$$

where $\varepsilon > 0$.
Case A implies $f(\gamma_0) > f(\alpha) > f(\gamma_1) \geq f(\gamma_2) .... \geq f(\gamma_{p-1}) \geq f(\gamma_p)$, and thus, $|V_\alpha| = 1$.
Case B implies $f(\alpha) > f(\gamma_0) \geq f(\gamma_1) \geq f(\gamma_2) .... \geq f(\gamma_{p-1}) \geq f(\gamma_p)$, and thus, $|V_\alpha| = 0$.
Hence, for each $\alpha^p \in K$ with $1 \leq p \leq d$, we have that $|V_\alpha| \leq 1$.

□

Filtration of the clique complex based on weights of critical simplices

Given a simplicial complex K, its dimension d and a discrete Morse function f on K, the algorithm 2 determines the weights of critical simplices in K. In the pseudocode of the algorithm 2, lines 2-6 initialize a variable `IsCritical`$[\alpha]$ associated to every simplex α in clique complex K to be True. Lines 7-17 determine the critical simplices in K by checking for the condition in equation 7 of the main text which defines a critical simplex. Lines 18-28 determine the weights of critical simplices or critical weights in K. Finally, the algorithm 2 outputs an array $w_c[]$ which contains an increasing sequence of critical weights in K. Subsequently, this increasing sequence of critical weights will be used for the filtration of the clique complex K.

Given an unweighted and undirected graph G, we restrict the construction of clique complex K by including simplices up to a maximum dimension d. Then, the algorithm 3 creates the filtration of clique complex K based on weights of critical simplices as described in the Theory section of the main text. In the pseudocode of the algorithm 3, lines 2-6 assigns a non-negative function g to 0-simplices in clique complex K. Line 7 calls the algorithm 1 for the assignment of weights satisfying discrete Morse function to every simplex in K. Line 8 calls the algorithm 2 to obtain an increasing sequence of unique weights corresponding to critical simplices in K. Lines 9-11 initialize a variable `IsAdded`$[\alpha]$ associated to every simplex α in K which tracks whether the simplex α has been added to the filtration or not. Lines 12-31 compute the filtration weight of each simplex α in K as described in the Theory section of the main text.

In SI Table S1, we describe the role of key variables which appear in algorithms 2 and 3.

Algorithm 2 Algorithm to compute the weights of critical simplices in K corresponding to the discrete Morse function f

```
function GetCriticalWeights(K, d, f)

    for p = 0, ···, d do                ▷ Initialize IsCritical variable associated with each simplex in K
        for each p-simplex α ∈ K do
            IsCritical[α]= True         ▷ IsCritical indicates whether a given simplex is critical or not
        end for
    end for

    for p = 1, ···, d do                ▷ Determine the critical simplices in K
        for each p-simplex α ∈ K do
            Let Faces[ ] be an array of all (p − 1)-dimensional faces of α
            Sort Faces[ ] such that f(Faces[i]) ≥ f(Faces[i+1]) for each i ∈ {0, 1, ···, p − 1}
            Let γ0 = Faces[0]
            if f(γ0) ≥ f(α) then
                IsCritical[α] = False
                IsCritical[γ0] = False
            end if
        end for
    end for

    Initialize i = 0
    Declare empty array wc[ ]
    for p = 0, ···, d do                ▷ Determine the weights of critical simplices in K
        for each p-simplex α ∈ K do
            if IsCritical[α] = True then
                wc[i] = f(α)
                i = i + 1
            end if
        end for
    end for
    Sort array wc[ ] in increasing order and remove duplicates

    return wc[ ]

end function
```

Algorithm 3 Algorithm to determine the filtration weights of the simplices in the clique complex of a simple graph G

K = d-dimensional clique complex of graph G

$deg_{max} =$ Maximum degree of a vertex in graph G
for each 0-simplex $\alpha \in K$ **do** ▷ Assign non-negative function g to 0-simplices in K
    $\varepsilon =$ random$(0, 0.5)$ ▷ ε is generated randomly during runtime using a uniform distribution on $(0, 0.5)$
    $g[\alpha]$ = deg_{max} - degree(α) + ε
end for

f = DISCETEMORSEFUNCTION(K, d, g) ▷ Call Algorithm 1
$w_c[\ ]$ = GETCRITICALWEIGHTS(K, d, f) ▷ Call Algorithm 2

for each simplex $\alpha \in K$ **do** ▷ Initialize `IsAdded` variable associated with each simplex in K
    `IsAdded`$[\alpha] = False$ ▷ `IsAdded` indicates whether a given simplex has been added to the filtration.
end for

for $i = 0, \cdots, len(w_c[\ ]) - 1$ **do** ▷ Calculate Filtration weight for each simplex in K
    for each simplex $\alpha \in K$ **do**
        if $f(\alpha) \leq w_c[i]$ AND `IsAdded`$[\alpha] = False$ **then**
            `FiltrationWeight`$[\alpha] = w_c[i]$
            `IsAdded`$[\alpha] = True$
            for each face $\gamma < \alpha$ **do**
                if `IsAdded`$[\gamma] = False$ **then**
                    `FiltrationWeight`$[\gamma]$ = $w_c[i]$
                    `IsAdded`$[\gamma] = True$
                end if
            end for
        end if
    end for
end for

for each simplex $\alpha \in K$ **do**
    if `IsAdded`$[\alpha] = False$ **then**
        `FiltrationWeight`$[\alpha] = w_c[len(w_c[\ ]) - 1]$
        `IsAdded`$[\alpha] = True$
    end if
end for

Computational aspects of our algorithms

Time complexity of Algorithm 1

We briefly discuss here some computational aspects of the algorithm 1 in the main text. Given a simplicial complex K, its dimension d and a non-negative real-valued function g on the 0-simplices of K, algorithm 1 assigns weights to any simplex in K, producing a discrete Morse function f on K.

We remark that the algorithms 1, 2 and 3 were implemented using the C++ programming language. The simplices of a d-dimensional simplicial complex were stored using the set container which is a part of the C++ Standard Template Library (STL). The association of the discrete Morse function values to the corresponding simplices was implemented using the map container of the C++ STL. Implementing the storage of the `Flag` variable associated with each simplex was also done using the map container of C++ STL. We remark that accessing a particular value of a map container has a complexity which is logarithmic with respect to the size of the container, namely, the number of simplices n.

Assuming that the dimension d of K is constant, we here present a brief discussion about the complexity of algorithm 1 with respect to the number of simplices n in K. We remark that since the dimension d is constant and the complexity of accessing values using map containers is logarithmic with respect to n, lines 2-9 in algorithm 1 have a complexity of $O(n \log n)$. The complexity of the operation corresponding to line 12 in algorithm 1, namely, finding the $(p-1)$ -dimensional faces of a p-simplex is independent of n. Such an operation depends on p which has an upper bound of d that has been assumed to be a constant.

The operation in line 13 of algorithm 1 corresponds to arranging the simplices computed in the preceding line based on their function values. As noted earlier, accessing values using map containers is logarithmic with respect to n. Thus, since $p \leq d$ and d is fixed, the operation represented by line 13 of algorithm 1 has a complexity of $O(\log n)$. Lines 14-22 of algorithm 1 except the random number generation in line 20 of algorithm 1 require accessing values of map containers. Random number generation based on a uniform distribution using C++ can be implemented with amortized constant complexity. Thus, the block of pseudocode represented by lines 14-22 in algorithm 1 have complexity of $O(\log n)$. Since d is constant and for any p the number of p-simplices is bounded by the total number of simplices n, the pseudocode represented in lines 10-24 of algorithm 1 has a complexity of $O(n \log n)$. Hence the complexity of algorithm 1 with respect to the number of simplices n can be described as $O(n \log n)$.

Time complexity of algorithms 1, 2 and 3 with respect to the number of critical simplices

Firstly, it is apparent that the complexity of algorithm 1 is independent of the number of critical simplices since the notion of critical simplices is relevant only after the discrete Morse function has been constructed.

Algorithm 2 accepts as inputs a simplicial complex K, its dimension d and a discrete Morse function f on K and outputs the sorted sequence of weights which correspond to the critical simplices in K. Let m denote the total number of critical simplices in K corresponding to f. We assume henceforth that the simplicial complex K and its dimension d are fixed. This implies that the number of simplices n and the dimension d are treated as constants. In such a situation, the complexity of all lines in the pseudocode of algorithm 2 except for line 28 is constant and independent of m. Line 28 of algorithm 2 represents sorting the array which contains the function values of all critical simplices, i.e., the critical weights, and removing any duplicate entries. The number of critical weights is less than or equal to the number of critical simplices m, and thus, the operation represented by line 28 of algorithm 2 can be implemented with a complexity of $O(m^2)$. Hence, assuming that the simplicial complex K and its dimension d are fixed, algorithm 2 can be implemented with a complexity of $O(m^2)$ in respect to the number of critical simplices m.

Algorithm 3 accepts a graph as input and determines the filtration weights of the simplices in the d-dimensional clique complex K of G. Similar to the analysis of algorithm 2, we assume that the graph G and the dimension d of K is fixed which implies that the number of simplices in the d-dimensional clique complex K of G is also constant. In such a situation, lines 1-7 of algorithm 3 have constant complexity which is independent of the number of critical simplices m. As seen in the preceding paragraph, provided K and d are fixed, the complexity of algorithm 2 and hence the complexity of line 8 of algorithm 3 is $O(m^2)$. Lines 9-11, lines 13-24 and lines 26-31 in the pseudocode of algorithm 3 have constant complexity which is independent of the number of critical simplices m. The number of times the operations of lines 13-24 in algorithm 3 are repeated is equal to the number of critical weights which is less than or equal to the number of critical simplices m. Thus, assuming that the graph G and the dimension d of the corresponding clique complex K are fixed, algorithm 3 can be implemented with a complexity of $O(m^2)$ in respect to the number of critical simplices m.

Theoretical results on stability of persistent homology and persistence diagrams of discrete Morse functions

Stability of persistent homology

In Algorithm 1, some choices are made in assigning values to every simplex in order to get a discrete Morse function. A natural question arises which is the following. Under what conditions do two discrete Morse functions give the same persistent homology groups. We remark that in Forman's theory[3], the actual values of the discrete Morse functions are less important than the *gradient vector field* that the discrete Morse function induces. For the definition of a gradient vector field, we refer the reader to Definition 3.3 in Forman's[3] article. In his expository paper[3], Forman asserts on page 15 that: *"In fact, this gradient vector field contains all of the information that we will need to know about the function for most applications."*

Note that two Morse functions f and g induce the same gradient vector field if and only if they satisfy the following condition (see Forman[4] or Theorem 3.1 in Ayala *et al.*[5]):

$$\mathbf{C0}: f(\alpha) < f(\beta) \Leftrightarrow g(\alpha) < g(\beta) \text{ for a } p\text{-simplex } \alpha \text{ and a } (p+1)\text{-simplex } \beta \text{ such that } \alpha^p < \beta^{p+1}$$

We show that for two discrete Morse functions to have the same filtration given by their level subcomplexes on critical weights, the actual values of the discrete Morse function are not as important as the *order relation* induced by the function on the simplices. The discrete Morse functions with the same filtration clearly give the same persistent homology groups. In particular, we show below that a sufficient condition for this to happen is that both functions have the same order relation with respect to the values assigned to the simplices.

Lemma 7. *Let f and g be two discrete Morse functions on a finite simplicial complex K. Assume that the following condition is satisfied:*

$$\mathbf{C1}: f(\alpha) \leq f(\beta) \Leftrightarrow g(\alpha) \leq g(\beta) \text{ for any pair of simplices } \alpha, \beta \in K.$$

Then f and g have the same set of critical simplices $C := \{c_1, c_2, \cdots, c_k\}$, and the level subcomplex of f corresponding to the critical cell c_i is the same as the level subcomplex of g corresponding to c_i, for any $i = 1, 2, \cdots, k$.

Proof. The condition **C1** guarantees that the functions f and g have the same gradient vector field, since **C1** implies **C0**. Therefore they have the same set of critical simplices C. Denote the level subcomplex of f (respectively, of g) corresponding to the critical cell c_i by $K^f(c_i)$ (respectively, by $K^g(c_i)$). Now, if $\alpha \in K^f(c_i)$, then there exists $\beta \in K$ such that $\alpha \leq \beta$ and $f(\beta) \leq f(c_i)$. Note that $\alpha \leq \beta$ represents that either two simplices α and β are same or α is a face of β. By assumption **C1** this implies that $g(\beta) \leq g(c_i)$, thus $\alpha \in K^g(c_i)$. So we get $K^f(c_i) \subseteq K^g(c_i)$. Reversing the roles of f and g in the above argument, we also get $K^g(c_i) \subseteq K^f(c_i)$. Thus $K^f(c_i) = K^g(c_i)$ for any critical cell $c_i \in C$. □

A simple example of two discrete Morse functions f and g which satisfy **C1** is the following. Starting with a discrete Morse function f obtain g by *sliding* the values of f by a fixed real number r. A slightly more sophisticated example of two functions which satisfy **C1** is the following. Given a discrete Morse function f on a finite simplicial complex K, one can arrange the values of f on the simplices of K in non-decreasing order. Suppose that α and β are simplices of K which are adjacent in this ordering, i.e., we have $f(\alpha) \leq f(\beta)$ and no other simplex σ exists such that $f(\sigma)$ lies in the interval $[f(\alpha), f(\beta)]$, and let us call such pairs of simplices (α, β) as *f-successive*. If for each f-successive pair of simplices (α, β), one adds a non-negative number $\delta(\alpha, \beta)$ to $f(\alpha)$ such that $\delta(\alpha, \beta) \leq f(\beta) - f(\alpha)$, we obtain a new discrete Morse function g given by $g(\alpha) := f(\alpha) + \delta(\alpha, \beta)$, for which any pair (α, β) is f-successive if and only if it is g-successive. It is easy to deduce that f and g then satisfy the condition **C1**. Thus, f and g will have the same persistent homology groups.

Stability of persistence diagrams

We remark that two discrete Morse functions may have the same filtration (and thus the same persistent homology groups) but their persistence diagrams may differ if their critical weights (i.e., values on the critical simplices) are different. A sufficient condition that assures that two discrete Morse functions f and g have the same persistence diagrams is the following, keeping the notations of Lemma 7:

C2: in addition to **C1**, we have $f(c_i) = g(c_i)$ for any $c_i \in C$

Even if the condition **C2** is not satisfied, there are stability theorems which give conditions under which the bottleneck distance between the respective persistence diagrams does not change much, i.e., the persistence diagrams are *close* in the bottleneck metric. The first such stability theorem was given by Cohen-Steiner *et al.*[6]. This result[6] is not directly applicable to discrete Morse functions, as they are not continuous. However, we show below that stability theorems for discrete Morse functions for finite regular CW-complexes can be inferred from stability results in Chazal *et al.*[7] (see also Bauer *et al.*[8]). For

simplicity we give the arguments for discrete Morse functions on a finite simplicial complex, although all the results below are also valid for finite regular CW-complexes.

Recall the ∞-Wasserstein distance or bottleneck distance between two multisets X and Y in $\mathbb{R}^2$:

$$W_\infty(X,Y) = \inf_{\eta:X\to Y} \sup_{x\in X} ||x-\eta(x)||_\infty. \tag{A.8}$$

In the above equation, the supremum is taken over all bijections $\eta : X \to Y$ (with the convention that a point with multiplicity $k \in \mathbb{N}$ is considered as k individual points) and for $(a,b) \in \mathbb{R}^2$, $||(a,b)||_\infty := \max\{|a|,|b|\}$.

For a discrete Morse function f with critical values $w_i := f(c_i), i = 1,2,\cdots,n$, the kth persistence diagram $D^k f$ (or simply Df when the value of k is fixed), is defined as follows. Consider the multiset of points $W_f^k := \{(w_i,w_j) : w_i < w_j, i,j = 1,2,\cdots,n\}$ with each point (w_i,w_j) endowed with the multiplicity $\mu_k(w_i,w_j)$ given by (see e.g. Di Fabio *et al.*[9]):

$$\mu_k(w_i,w_j) := \lim_{\varepsilon\to 0^+} (\beta_{w_i+\varepsilon}^{w_j-\varepsilon} - \beta_{w_i+\varepsilon}^{w_j+\varepsilon} + \beta_{w_i-\varepsilon}^{w_j+\varepsilon} - \beta_{w_i-\varepsilon}^{w_j-\varepsilon})$$

where $\beta_x^y := \mathrm{rank}(H_k(K^f(x)) \to H_k(K^f(y)))$ for $x,y \in \mathbb{R}$ with $x < y$. Denote by Δ the diagonal in $\mathbb{R}^2$ considered as a multiset with infinite multiplicity given to each of its points.

Definition 8. *The persistence diagram $D^k f$ is the subset of $W_f^k \cup \Delta$ consisting of points (u,v) with $\mu_k(u,v) > 0$.*

We state below the stability results from Chazal *et al.*[7] that we shall need, and to do so, we recall the concepts of *persistence modules* and *ε-interleaving*:

Definition 9. (Persistence modules (Definition 2.2. in Chazal *et al.*[7])) *Let R be a commutative ring with unity. A collection $\mathscr{F} = \{F^\alpha\}_{\alpha\in\mathbb{R}}$ of R-modules F^α together with homomorphisms $\phi_{\alpha\beta} : F^\alpha \to F^\beta$ for $\alpha \le \beta$, is called a persistence module if*

$$\forall \alpha \le \beta \le \gamma, \qquad \phi_{\alpha\alpha} = \mathrm{id}_{F^\alpha} \text{ and } \qquad \phi_{\alpha\beta} \circ \phi_{\beta\gamma} = \phi_{\alpha\gamma}$$

A persistence module is called δ-tame if for any $\alpha < \alpha + \delta < \beta$ one has $\mathrm{rank}\,\phi_{\alpha\beta} < \infty$.

Definition 10. (ε-interleaving of functions (Definition 4.1 in Chazal *et al.*[7])) *Let $\varepsilon > 0$, and let f and g be two real-valued functions on a finite simplicial complex K. For $x \in \mathbb{R}$, denote the sub-level sets $f^{-1}(-\infty,x]$ of f (respectively, g) by F^x (respectively, G^x). The functions f and g are said to be strongly ε-interleaved if for any $a \in \mathbb{R}$ and $n \in \mathbb{Z}$, one has*

$$F^{a+2n\varepsilon} \subseteq G^{a+(2n+1)\varepsilon} \subseteq F^{a+(2n+2)\varepsilon}$$

The main result for ε-interleaved functions that we shall need is as follows:

Lemma 11. (Lemma 4.2(iii) in Chazal *et al.*[7]) *Given $\varepsilon > 0$, two functions f and g are strongly ε-interleaved if and only if $||f-g||_\infty < \varepsilon$.*

Definition 12. (ε-interleaving of persistence modules (Definition 4.3 in Chazal *et al.*[7])) *Let $\mathscr{F} = \{F^\alpha\}_{\alpha\in\mathbb{R}}$ and $\mathscr{G} = \{G^\alpha\}_{\alpha\in\mathbb{R}}$ be persistence modules over a commutative unital ring R. Let $\varepsilon > 0$, then $\mathscr{F}$ and $\mathscr{G}$ are said to be strongly ε-interleaved if for all $a \in \mathbb{R}$ and $n \in \mathbb{Z}$, there exist homomorphisms $\Phi_{a+2n\varepsilon} : F^{a+2n\varepsilon} \to G^{a+(2n+1)\varepsilon}$ and $\Psi_{a+(2n+1)\varepsilon} : G^{a+(2n+1)\varepsilon} \to F^{a+(2n+2)\varepsilon}$ such that the following diagram commutes:*

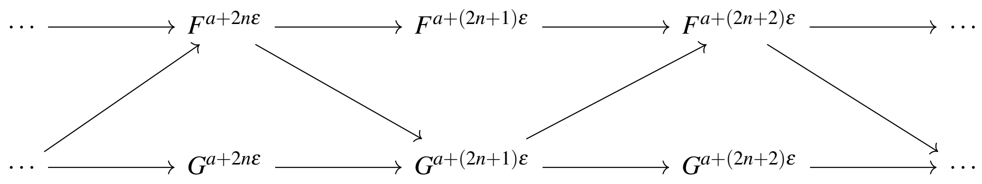

If f and g are strongly ε-interleaved, then the associated persistence modules given by their k^{th} singular homology groups are also strongly ε-interleaved for any $k \in \mathbb{N} \cup \{0\}$, the associated homomorphisms in Definition 10 are induced by the corresponding inclusion maps. Note that these persistence modules are 0-tame since the functions are defined on a finite simplicial complex K.

One can define persistence diagrams for δ-tame persistence modules (see Definitions 3.6 and 3.10 in Chazal *et al.*[7]). Denote the persistence diagram of a δ-tame persistence module $\mathscr{F}$ by $D_\delta\mathscr{F}$. The persistence diagram of persistence modules generalizes the classical persistence diagrams associated with real-valued functions, i.e., if f is a tame real-valued function on a finite simplicial complex K, the associated 0-tame persistence module given by the kth singular homology groups of its sublevel sets F^α, denoted $\mathscr{F}_k := \{H_k(F^\alpha)\}_{\alpha\in\mathbb{R}}$, has the same persistence diagram as the kth persistence diagram of f, i.e. $D^k f = D_0\mathscr{F}_k$ (see Remark 1, page 12 in Chazal *et al.*[7] for a short proof).

The next lemma shows that if f is a discrete Morse function and $K^f(\alpha), \alpha\in\mathbb{R}$ are the level subcomplexes of f, then the persistence diagram of the 0-tame persistence module $\mathscr{K}_k = \{H_k(K^f(\alpha))\}_{\alpha\in\mathbb{R}}$ is the same as the kth persistence diagram $D^k f$ of f corresponding to the filtration of K given by the level subcomplexes at critical weights, i.e., $D^k f = D_0\mathscr{K}_k$. The proof is essentially the same as the arguments given in Remark 1, page 12 of Chazal *et al.*[7], except that we use Forman's Lemma 2.6[3] for comparing homology groups across critical weights. Note that due to Forman's Lemma 2.6[3], the set of k-homological critical values of f, i.e., points $c\in\mathbb{R}$ such that for any $\varepsilon>0$ the homomorphism on the k-homology groups induced by inclusion map $K^f(c-\varepsilon)\hookrightarrow K^f(c+\varepsilon)$ is *not* an isomorphism, is a subset of its critical weights in general. However, it is easy to see that only those points (w_i,w_j) appear in the persistence diagram of f for which both w_i and w_j are k-homological critical values of f (otherwise the multiplicity of such points is zero).

Lemma 13. *We have* $D^k f = D_0\mathscr{K}_k$.

Proof. Let k be fixed, to simplify the notation we denote the k-persistence diagrams of f and $\mathscr{K}_k$ by Df and $D\mathscr{K}$, respectively. From the definition of the persistence diagrams of persistence modules (Definition 3.6 in Chazal *et al.*[7]), it suffices to show that there exists $\varepsilon>0$ $x\in(0,\varepsilon)$, such that $W_\infty(Df, D\mathscr{K}_{\frac{\varepsilon}{2^n},x}) < \varepsilon/2^n$ for any $n\geq 1$, where $D\mathscr{K}_{\frac{\varepsilon}{2^n},x}$ is the multi-subset of $\mathbb{R}^2$ given by points of non-zero multiplicity in the union of the grid

$$G_{\varepsilon/2^n,x} := \{(x+\frac{m\varepsilon}{2^n}, x+\frac{m'\varepsilon}{2^n})\in\mathbb{R}^2, m,m'\in\mathbb{Z} : m'>m\}$$

and the diagonal Δ, where the multiplicity $\mu(m,m')$ of a point $(x+\frac{m\varepsilon}{2^n}, x+\frac{m'\varepsilon}{2^n})\in G_{\varepsilon/2^n,x}$ is given by the following formula from Definition 3.1(ii) in Chazal *et al.*[7]:

$$\begin{aligned}\mu(m,m') \quad &:= \quad \mathrm{rank}(H(m)\to H(m'-1)) - \mathrm{rank}(H(m)\to H(m'))\\ &+ \quad \mathrm{rank}(H(m-1)\to H(m')) - \mathrm{rank}(H(m-1)\to H(m'-1))\end{aligned}$$

where we have used the notation $H(m) := H_k(K^f(x+\frac{m\varepsilon}{2^n})), m\in\mathbb{Z}$ for simplicity.

Since the number of critical weights is finite, one can choose $\varepsilon>0$, $x\in(0,\varepsilon]$ such that none of the grids $G_{\varepsilon/2^n,x}$ have a point outside the diagonal that coincides with a point in Df. Then, for each $n\geq 1$ a point $(w_i,w_j)\in Df$ is contained in exactly one of the cells C in the grid $G_{\varepsilon/2^n,x}$. It is easy to check using Forman's Lemma 2.6[3] that the multiplicity $\mu(w_i,w_j)$ is then equal to the multiplicity of the upper-right corner of C, while all other off diagonal points in $D\mathscr{K}$ which are not upper-right corners of cells containing a point of Df, have multiplicity zero. Therefore the bijection $\Phi_{\varepsilon,n} : Df\to D\mathscr{K}_{\varepsilon/2^n,x}$ which maps (w_i,w_j) in Df to the upper right corner of the unique cell in $G_{\varepsilon/2^n,x}$ containing (w_i,w_j) and any point in the region $\{(x,y)\in\mathbb{R}^2 : y\in[x,x+\varepsilon/2^n]\}$ to the nearest point on the diagonal, moves points by a distance at most $\frac{\varepsilon}{2^n}$. Thus, we get $W_\infty(Df, D\mathscr{K}_{\frac{\varepsilon}{2^n},x}) < \varepsilon/2^n$ for any $n\geq 1$. □

We can now state the stability theorem for ε-interleaved persistence modules:

Theorem 14. (Theorem 4.4 in Chazal *et al.*[7]) *Let $\mathscr{F}$ and $\mathscr{G}$ be two strongly ε-interleaved persistence modules which are δ-tame for some $\delta\geq 0$. Then,*

$$W_\infty(D_\delta\mathscr{F}, D_\delta\mathscr{G}) < 3\varepsilon.$$

We are now ready to state a stability theorem for persistence diagrams for discrete Morse functions:

Theorem 15. *Let f and g be discrete Morse functions on a finite simplicial complex K which induce k^{th} persistence diagrams $D^k f$ and $D^k g$, respectively. Suppose that there exists $\varepsilon>0$, such that $||f-g||_\infty<\varepsilon$. Then,*

$$W_\infty(D^k f, D^k g) < 3\varepsilon.$$

Proof. Let us show that the level subcomplexes of f and g are strongly ε-interleaved. Indeed, since $||f-g||_\infty<\varepsilon$, f and g are strongly ε-interleaved by Lemma 11. Let $c\in\mathbb{R}$ and let $\sigma\in K^f(c)$ and denote by F^c the sub-level set $f^{-1}((-\infty,c])$ of f; G^c is

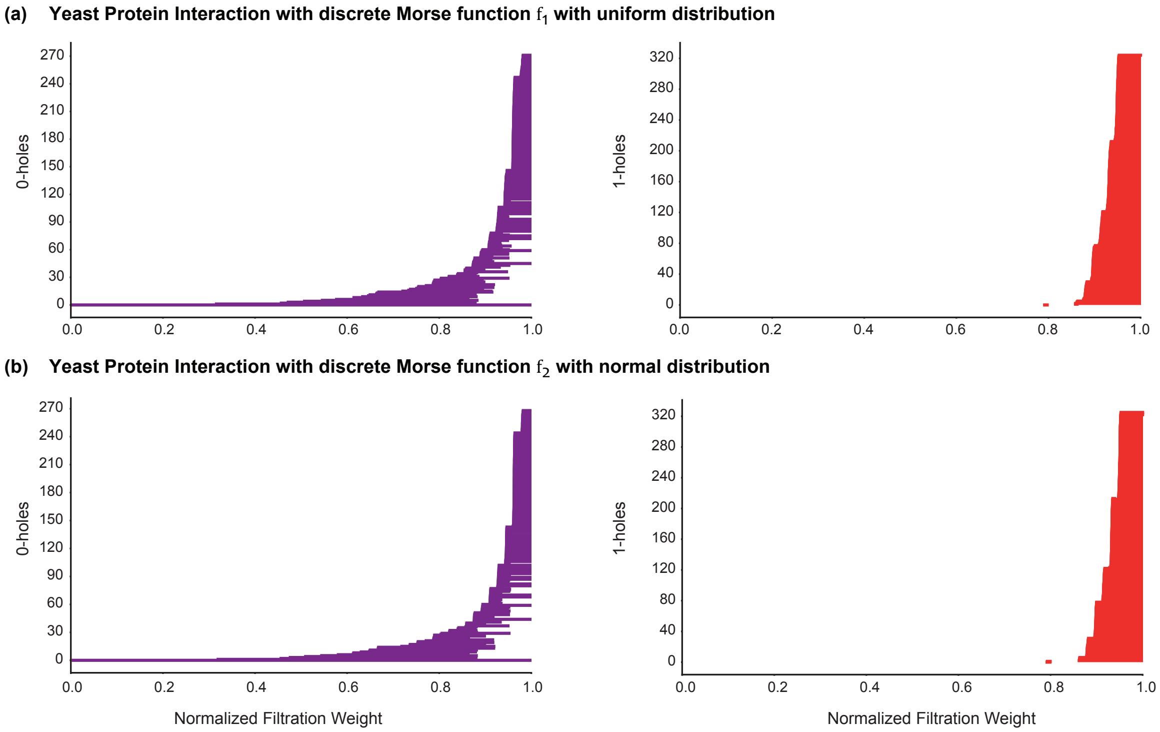

Figure A1. Barcode diagrams for H_0 and H_1 in the Yeast protein interaction network computed using two discrete Morse functions f_1 and f_2 generated by making small variations in Algorithm 1. f_1 uses a uniform distribution on $[0.1, 0.4)$ for generating the values of ε in Algorithm 1, f_2 uses a normal distribution with mean $\mu = 0.25$ and standard deviation $\sigma = 0.05$ for generating the values of ε in Algorithm 1.

defined similarly for the discrete Morse function g. Then, by Lemma 3.2 of Forman[10], there exists a co-face τ of σ such that $f(\tau) \leq c$, i.e. $\tau \in F^c$. By the strong ε-interleaving property, one gets $F^c \subseteq G^{c+\varepsilon}$, so we have $g(\tau) \leq c + \varepsilon$, which implies that $\sigma \in K^g(c+\varepsilon)$, so that we get $K^f(c) \subseteq K^g(c+\varepsilon)$. Similarly one easily gets $K^g(c) \subseteq K^f(c+\varepsilon)$ for any $c \in \mathbb{R}$. This implies that the persistence modules of f and g given by the kth homology groups of the level subcomplexes are strongly ε-interleaved for any $k \in \mathbb{N} \cup \{0\}$. Hence by Theorem 14, one gets:

$$W_\infty(D^k f, D^k g) < 3\varepsilon$$

This concludes the proof. □

As a concluding remark to this section, we observe that the bound of 3ε can be improved to ε, following the same arguments as given in the proof of Theorem 4.8 in Chazal *et al.*[7], we omit the details here.

Illustration of the stability of persistence diagrams using a real network example

As an illustration of the stability property, we compute the bottleneck distance between persistence diagrams of two discrete Morse functions f_1 and f_2 on the Yeast protein interaction network[11] generated by making small variations in Algorithm 1. The construction of f_1 uses a uniform distribution on $[0.1, 0.4)$ for generating the values of ε in Algorithm 1, while the construction of f_2 uses a normal distribution with mean $\mu = 0.25$ and standard deviation $\sigma = 0.05$ for generating the values of ε in Algorithm 1. The L^∞-norm of the difference $||f_1 - f_2||_\infty$ is 18.3782, while the bottleneck distance between their *total* persistence diagrams (i.e., the persistence diagram of the persistence module $\mathscr{K}_* := \{\oplus_{k=0}^{\dim K} H_k(K^f(\alpha))\}_{\alpha \in \mathbb{R}}$), is computed to be $W_\infty(Df_1, Df_2) = 0.0061$. The barcode diagrams for the 0th and 1st homology groups in the Yeast protein interaction network[11] with the two discrete Morse functions, f_1 and f_2, are shown in Appendix figure A1.

References

1. Munkres, J. Elements of algebraic topology (CRC Press, 2018).

2. Dummit, D. & Foote, R. Abstract algebra (Wiley, 2003), 3 edn.

3. Forman, R. A user's guide to discrete morse theory. Sém. Lothar. Comb. **48**, 1–35 (2002).

4. Forman, R. Some applications of combinatorial differential topology. In Lyubich, M. & Takhtajan, L. (eds.) Graphs and patterns in mathematics and theoretical physics, 73, 281–313 (American Mathematical Society (AMS), 2005).

5. Ayala, R., Fernández, L. M. & Vilches, J.A., Characterizing equivalent discrete Morse functions, Bull. Braz. Math. Soc. New Ser. **40 (2)**, 225–235 (2009).

6. Cohen-Steiner, D., Edelsbrunner, H. & Harer, J. Stability of persistence diagrams. Discret. & Comput. Geom. **37**, 103–120 (2007).

7. Chazal, F., Cohen-Steiner, D., Guibas, L. J. & Oudot, S., Stability of persistence diagrams revisited, INRIA Research report RR-6568 available at: https://hal.inria.fr/inria-00292566v1/ (2008).

8. Bauer, U., Lange, C. & Wardetzky, M., Optimal Topological Simplification of Discrete Functions on Surfaces, Discret. & Comput. Geom. **47(2)**, 347–377 (2012).

9. Di Fabio, B. & Ferri, M. Comparing Persistence Diagrams Through Complex Vectors. In Image Analysis and Processing — ICIAP 2015, 294–305 (Springer International Publishing, Cham, 2015).

10. Forman, R. Morse theory for cell complexes. Adv. Math. **134**, 90–145 (1998).

11. Jeong, H., Mason, S. P., Barabási, A. L. & Oltvai, Z. N. Lethality and centrality in protein networks. Nature **411**, 41–42 (2001).
