# Supplementary material for: Persistent homology of unweighted complex networks via discrete Morse theory
Source: Sci Rep. 2019 Sep 25;9:13817. doi: 10.1038/s41598-019-50202-3 (PMC6761140; doi:10.1038/s41598-019-50202-3)
Supplement: Supplementary file 2 — Supplementary Figures [file 41598_2019_50202_MOESM2_ESM.pdf]

Supplementary Figures
for
Persistent homology of unweighted complex networks via discrete Morse theory

Harish Kannan[1], Emil Saucan[2,3], Indrava Roy[1,*], and Areejit Samal[1,4,*]

[1]The Institute of Mathematical Sciences (IMSc), Homi Bhabha National Institute (HBNI), Chennai 600113 India
[2]Department of Applied Mathematics, ORT Braude College, Karmiel 2161002 Israel
[3]Department of Electrical Engineering, Technion, Israel Institute of Technology, Haifa 3200003 Israel
[4]Max Planck Institute for Mathematics in the Sciences, Leipzig 04103 Germany
[*]indrava@imsc.res.in; asamal@imsc.res.in

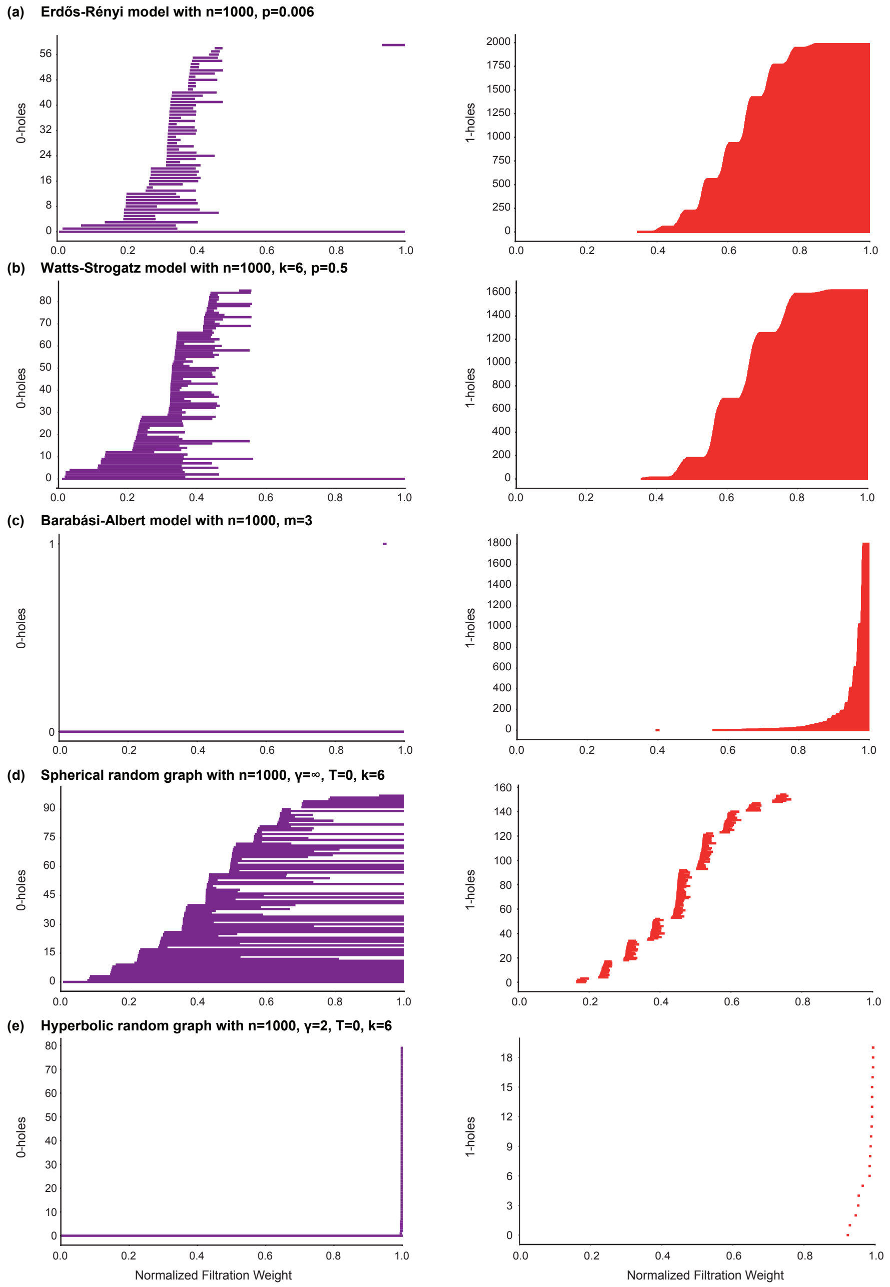

Figure S1. Barcode diagrams for H_0 and H_1 in model networks with expected average degree 6.

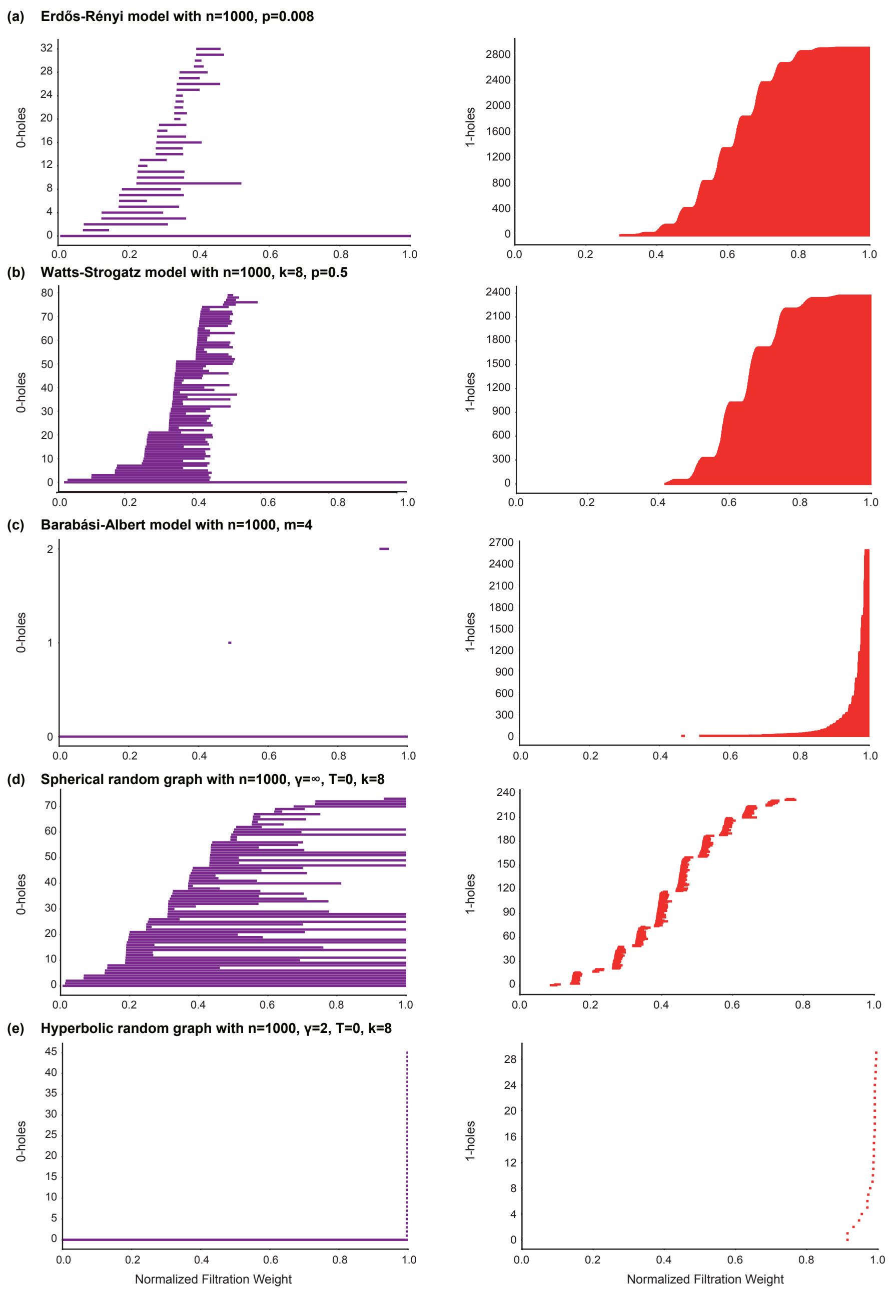

Figure S2. Barcode diagrams for H_0 and H_1 in model networks with expected average degree 8.

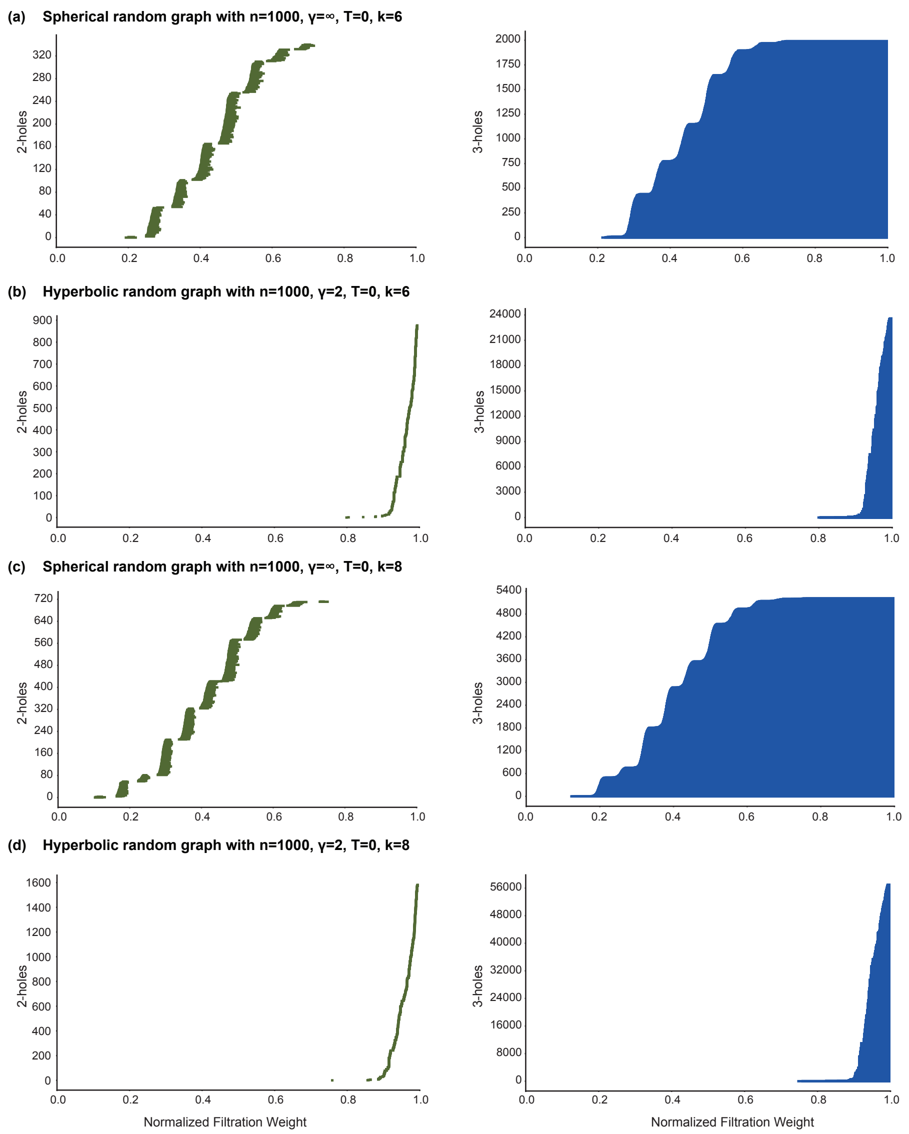

Figure S3. Barcode diagrams for H_2 and H_3 in model networks with expected average degree 6 and 8. (a) Spherical random graphs with expected average degree 6. (b) Hyperbolic random graphs with expected average degree 6. (c) Spherical random graphs with expected average degree 8. (d) Hyperbolic random graphs with expected average degree 8.

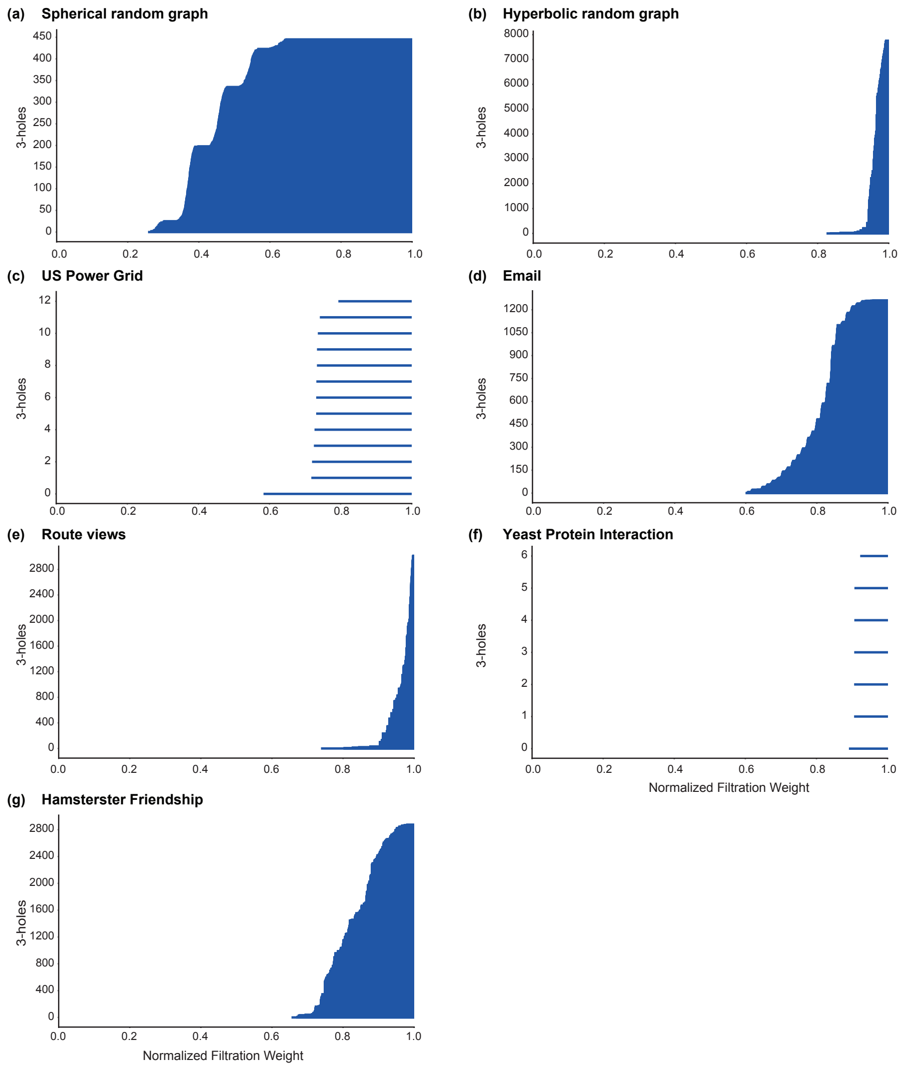

Figure S4. Barcode diagrams for H_3 in model and real networks. (a) Spherical random graphs produced from HGG model with $n = 1000$, $T = 0$, $k = 4$ and $\gamma = \infty$. (b) Hyperbolic random graphs produced from HGG model with $n = 1000$, $T = 0$, $k = 4$ and $\gamma = 2$. (c) US Power Grid. (d) Email communication. (e) Route views. (f) Yeast protein interaction. (g) Hamsterster friendship.

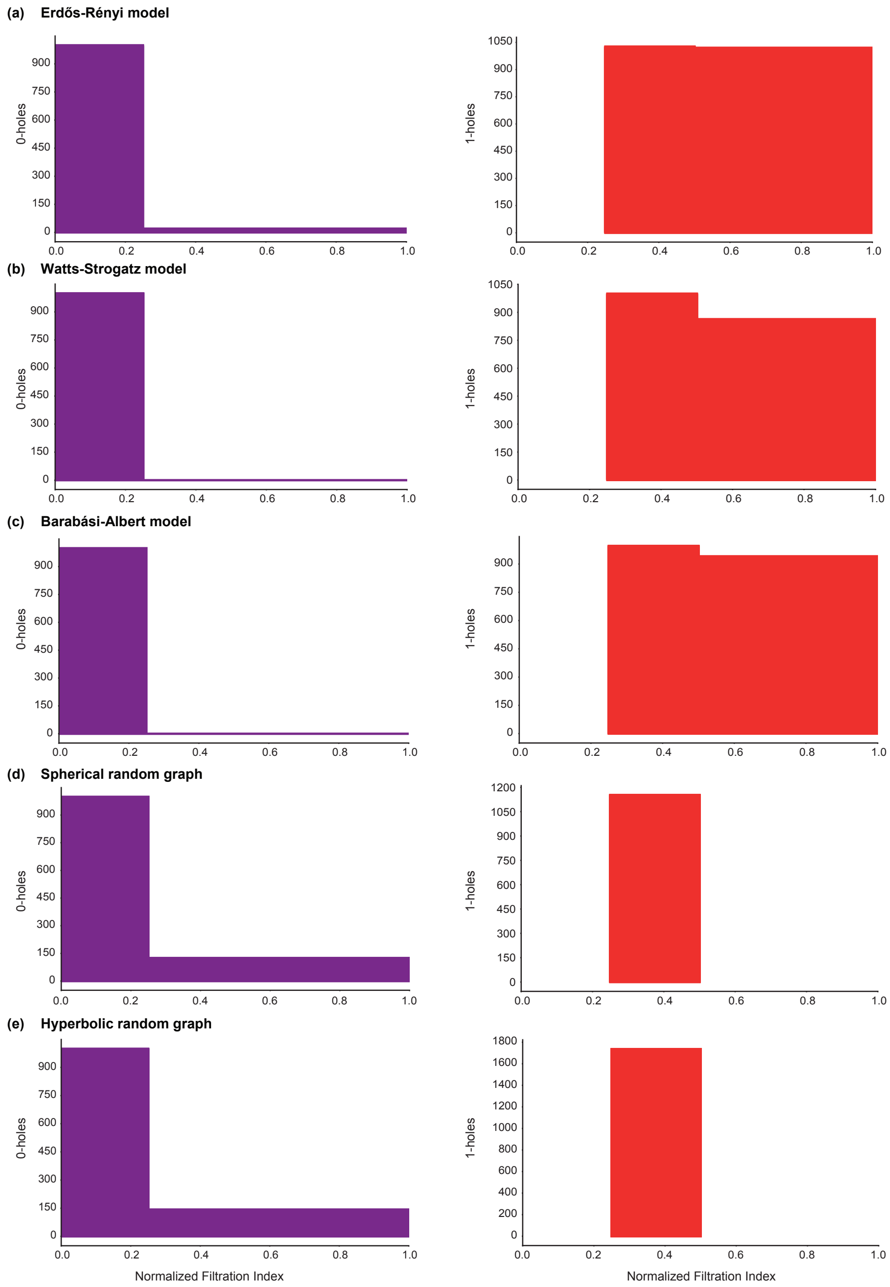

Figure S5. Barcode diagrams for H_0 and H_1 in model networks with expected average degree 4 obtained using the dimensional filtration scheme used in Horak *et al.* We restrict our investigation to the three-dimensional clique complex while computing these barcode diagrams for model networks using the dimensional filtration scheme of Horak *et al.* In this figure, we normalize the filtration index to be in the range 0 to 1, and p-holes with normalized filtration index 1 indicate that they never die. (a) ER model with $n = 1000$ and $p = 0.004$. (b) WS model with $n = 1000$, $k = 4$ and $p = 0.5$. (c) BA model with $n = 1000$ and $m = 2$. (d) Spherical random graphs produced from HGG model with $n = 1000$, $T = 0$, $k = 4$ and $\gamma = \infty$. (e) Hyperbolic random graphs produced from HGG model with $n = 1000$, $T = 0$, $k = 4$ and $\gamma = 2$.

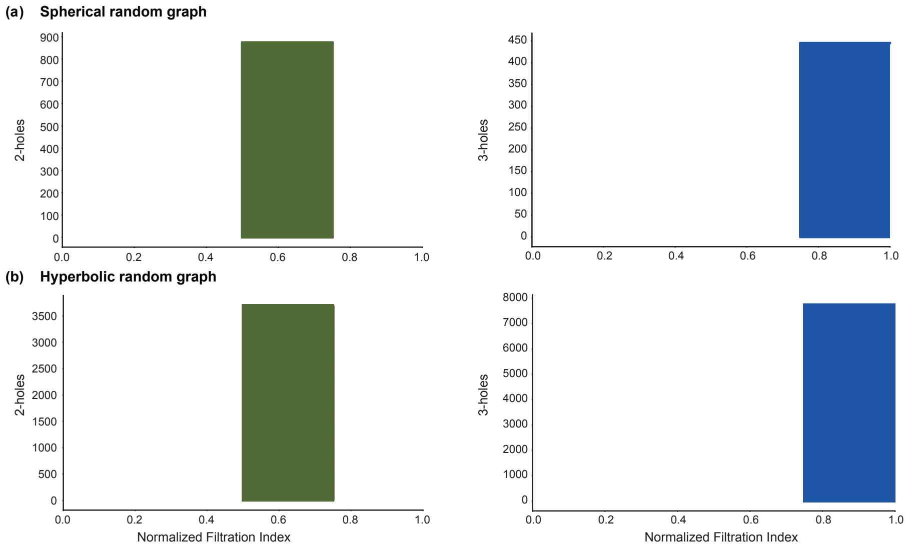

Figure S6. Barcode diagrams for H_2 and H_3 in model networks with expected average degree 4 obtained using the dimensional filtration scheme used in Horak *et al.* (a) Spherical random graphs produced from HGG model with $n = 1000$, $T = 0$, $k = 4$ and $\gamma = \infty$. (b) Hyperbolic random graphs produced from HGG model with $n = 1000$, $T = 0$, $k = 4$ and $\gamma = 2$.

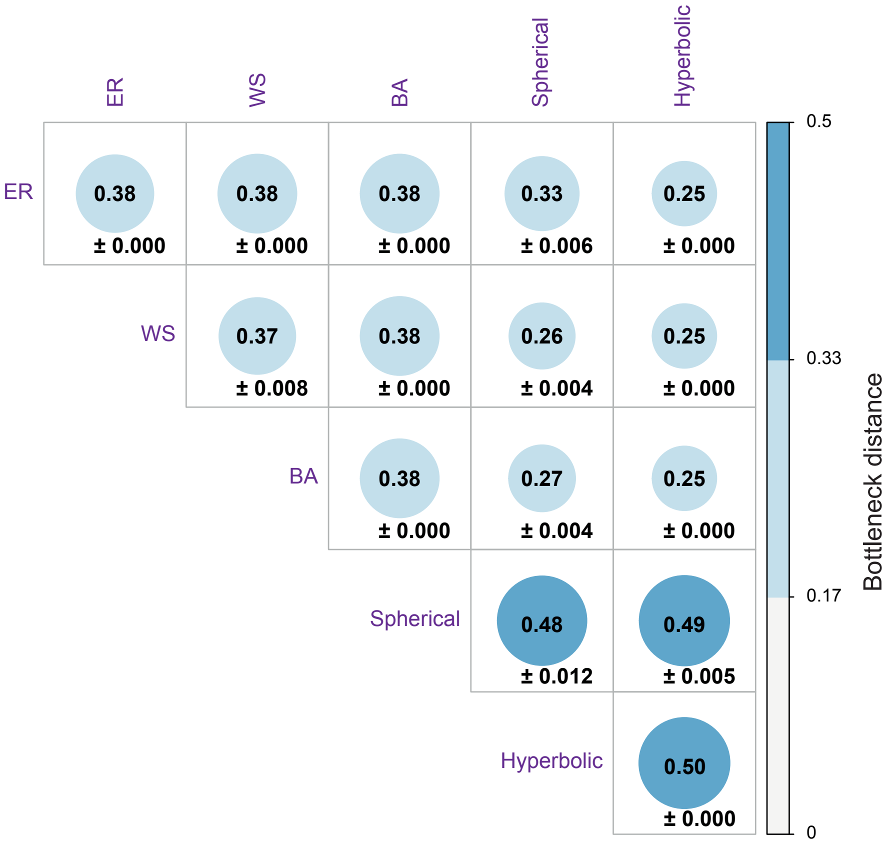

Figure S7. Bottleneck distance between persistence diagrams obtained using the dimensional filtration scheme used in Horak *et al.* for model networks, namely, ER model with $n = 1000$ and $p = 0.004$, WS model with $n = 1000$, $k = 4$ and $p = 0.5$, BA model with $n = 1000$ and $m = 2$, Spherical random graphs produced from HGG model with $n = 1000$, $T = 0$, $k = 4$ and $\gamma = \infty$, and Hyperbolic random graphs produced from HGG model with $n = 1000$, $T = 0$, $k = 4$ and $\gamma = 2$. For each of the five model networks, 10 random samples are generated by fixing the number of vertices n and other parameters of the model. We report the distance (rounded to two decimal places) between two different models as the average of the distance between each of the possible pairs of the 10 sample networks corresponding to the two models along with the standard error.
